# Exploring the emerging trends and hot topics of 5G technology application in wireless medicine: A bibliometric and visualization analysis

**DOI:** 10.1097/MD.0000000000043310

**Published:** 2025-07-18

**Authors:** Yunfan He, Jun Xie, Zheyang Weng, Fangyu Yang, Yutao Wei, Jun Liang, Jianbo Lei

**Affiliations:** a School of International Relations and Public Affairs, Fudan University, Shanghai, China; b National Institute of Intelligent Evaluation and Governance, Fudan University, Shanghai, China; c Information Technology Center, West China Hospital of Sichuan University/Engineering Research Center of Medical Information Technology, Ministry of Education, Chengdu, China; d Department of Clinical Engineering, Sir Run Run Shaw Hospital, School of Medicine, Zhejiang University, Hangzhou, Zhejiang Province, China; e School of Nursing, Capital Medical University, Beijing, China; f Health Science Center, Institute of Medical Technology, Peking University, Beijing, China; g Department of AI and IT, Second Affiliated Hospital, School of Medicine, Zhejiang University, Hangzhou, Zhejiang Province, China; h Center for Health Policy Studies, School of Public Health, Zhejiang University, Hangzhou, Zhejiang Province, China; i School of Medical Technology and Information Engineering, Zhejiang Chinese Medical University, Hangzhou, Zhejiang Province, China; j Key Laboratory of Cancer Prevention and Intervention, China National Ministry of Education, School of Medicine, Zhejiang University, Hangzhou, Zhejiang Province, China; k Clinical Research Center, The Affiliated Hospital of Southwest Medical University, Luzhou, Sichuan Province, China; l School of Medical Informatics and Engineering, Southwest Medical University, Luzhou, Sichuan Province, China; m Center for Medical Informatics, Health Science Center, Peking University, Beijing, China.

**Keywords:** 5G technology, hot topics, mobile health, smart hospital, telemedicine, wearable device

## Abstract

**Background:**

The online diagnosis and treatment model based on 5th generation mobile communication (5G) technology is one of the important ways to solve the imbalance between supply and demand of medical services.

**Objective:**

We systematically summarized Chinese and English literature on the application of 5G technology in the field of wireless medical and conducted a literature feature analysis.

**Methods:**

We used bibliometrics to quantitatively analyze the research trends and hot topics and comparatively analyzed the differences between research in China and other countries.

**Results:**

This study analyzed 1344 articles and found that China provided the most funding (531 [75.32%]) and far outnumbered other countries in this field (1014 vs 330), but the quality of articles and effective collaboration between authors need to be improved. The hot topics in this field have gradually shifted from the construction of 5G internet hospitals during the COVID-19 to the construction of smart hospitals based on the Internet of Medical Things, and the research focus has gradually shifted from the data transmission layer such as wearable devices to the application layer of smart medical services.

**Conclusion:**

Researchers can further refine the specific application of 5G technology in the field of wireless medical from the 3 major areas of the smart hospital system.

## 1. Introduction

The traditional medical model cannot meet people’s consumption demand for medical services, and COVID-19 has further exacerbated the imbalance between supply and demand of medical services. The traditional medical model, which focuses on offline consultations by doctors, is inefficient and supply-effective, and cannot meet people’s demand for efficient and high-quality medical services.^[1]^ The imbalance between supply and demand of medical services^[2]^ has also become increasingly prominent with the irrational allocation of medical resources^[3]^ and the increase in patients’ demand for high-quality medical services.^[4]^ During COVID-19, as a large amount of medical resources were prioritized for fighting the pandemic, the supply of offline medical services was further reduced, while the demand for public medical services surged, further intensifying the contradiction.^[5]^

5G technology is an important part of new infrastructure construction. The online diagnosis and treatment model based on 5G technology can help expand medical supply, improve service efficiency and quality, and reduce medical expenses. It is one of the important ways to solve the imbalance between the supply and demand of medical services. 5G technology refers to a new generation of broadband mobile communication technology with high-speed, low-latency, and large connection characteristics. It is the network infrastructure that realizes the interconnection of humans, machines, and things.^[6]^ As an important part of new infrastructure construction, 5G technology is strongly supported by the policies of many countries, such as China.^[7,8]^ Its high-speed data transmission can realize real-time early warning of wearable devices, remote surgery without delay, and the connection of everything in smart hospitals. With the rapid development of computer technology and information communication technology, through emerging technologies such as 5G technology, Internet of Things, cloud computing platform, and mobile health, the traditional medical model is deeply integrated with new information technology, gradually shifting from offline consultation to online integration diagnosis and treatment.^[9]^ Online diagnosis and treatment models such as telemedicine, online consultation, and internet hospitals^[10]^ have expanded the medical service platform, provided more high-quality medical service resources, improved medical efficiency, reduced medical expenses, and alleviated the imbalance between supply and demand of medical services^[11]^ and the impact of the COVID-19 on the traditional offline diagnosis and treatment model.^[12]^

At present, a large number of studies have focused on the application of 5G technology in different medical scenarios, and some reviews have systematically summarized the research status of 5G technology in a specific medical scenario. 5G technology is widely used in different medical scenarios. For example, Gupta et al^[13]^ used 5G technology in a remote surgery platform, which reduced data latency and storage costs and improved the accuracy and safety of remote surgery. Oleshchuk and Fensli^[14]^ proposed a technical solution for 5G remote patient monitoring, which strengthened the information transmission between patients and medical institutions. Huang et al^[15]^ designed a control framework for epidemic prevention and control based on 5G base station data, improving the real-time transmission and summary of epidemic data. In addition, some reviews have summarized the current application status of 5G technology in a certain medical field. For example, Guo and Li^[16]^ systematically summarized the application status and challenges of 5G technology in personalized care through literature content analysis. Li et al^[17]^ systematically summarized the research on the application of 5G technology in ophthalmic telemedicine and summarized the research progress and future directions. Ji and Yu^[18]^ analyzed the research status of 5G emergency medical services based on the characteristics, architecture, and key technologies of 5G emergency medical services.

However, previous related research was limited to the analysis of English literature, lacking a systematic review and summary of the application status of 5G technology in the field of wireless medical and lacking quantitative analysis in this field. Specifically, for breadth of analysis, the summarized literature is not comprehensive and is limited to analysis of English literature, lacking analysis of China, a country with a large population. For analysis perspective, previous studies have focused on a specific medical scenario and lacked attention to the entire medical field, making it impossible to understand the current application status and future direction of 5G technology in the medical industry. For analysis methods, most studies are limited to qualitative content analysis and lack the use of quantitative analysis methods, such as bibliometrics, to systematically describe research progress and hot topics in this field.

Therefore, this study aims to summarize the Chinese and English literature on the application of 5G technology in the medical field as of August 4, 2023, and use bibliometric methods to quantitatively analyze the latest research progress and topic trends in this field, making up for the shortcomings of traditional reviews. On the one hand, it helps the academic community grasp the publishing trends, core author groups, and hot topics in this field. On the other hand, it also provides feedback to functional departments and policymakers to better promote the development of 5G technology in the medical field.

## 2. Materials and methods

### 2.1. Ethical approval

As this article is a bibliometric study, with data sourced from publicly available literature databases, and does not involve human or experimental data, ethical approval is not required.

### 2.2. Data sources and retrieval strategies

In order to cover comprehensive and complete literature, we simultaneously searched 7 literature databases (including 3 Chinese databases: CNKI, Wanfang, and VIP; 4 English databases: Web of Science Core Collection, PubMed, Embase, and IEEE).^[19]^ We referred to the keywords of related research,^[20–23]^ MeSH terms in PubMed, and the research theme to construct the retrieval formula “(5G OR ‘5th generation mobile communication technology’ OR ‘5th generation mobile network’ OR ‘5th generation wireless system’ OR ‘5th-generation’) AND (medicine OR medical OR witmed OR ‘wise information technology of med’ OR ‘smart hospital’ OR ‘internet hospital’ OR ‘hospital informatization’ OR ‘hospital management’ OR ‘mobile health’ OR ‘digital health’ OR ‘wearable device’)” (for specific search terms and search results of different literature databases, see Table A1, Supplemental Digital Content, https://links.lww.com/MD/P388). We searched the 7 literature databases for literature related to the application of 5G technology in the medical field until August 4, 2023. To ensure the recall of the search, we conducted cross validation. Finally, a total of 6012 articles were retrieved, and 4880 articles remained to be screened after deduplication.

### 2.3. Data filtering

**Figure 1.**
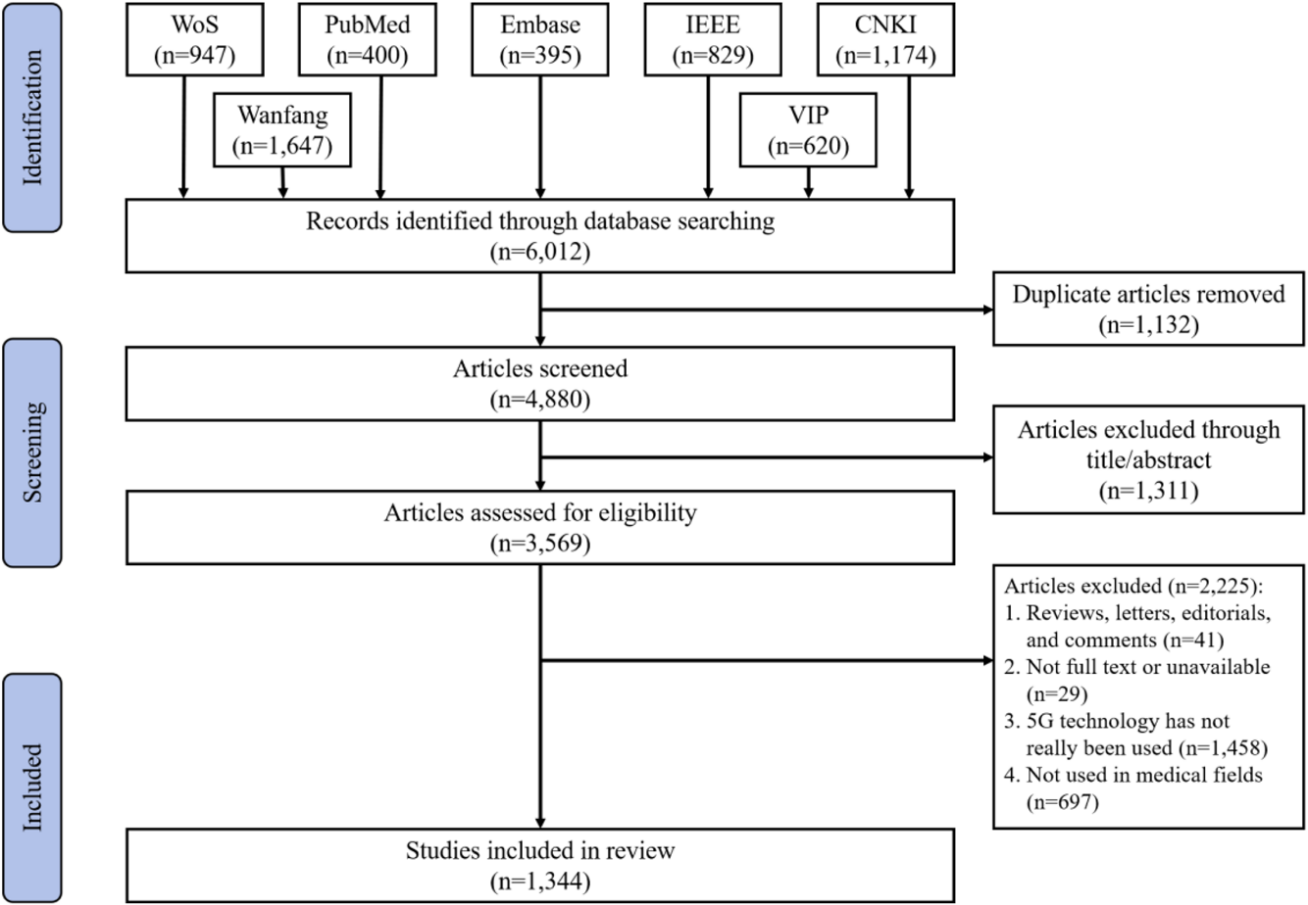
Literature screening flowchart.

This study strictly followed the Preferred Reporting Items for Systematic Reviews and Meta-Analyses (PRISMA) 2020 framework^[24]^ to complete the screening and analysis of the literature. We referred to previous studies^[21]^ and formulated the inclusion and exclusion criteria for this study (as shown below). Based on the inclusion and exclusion criteria, we first screened the literature through titles and abstracts, excluding 1311 articles, and then rescreened the full text, further excluding 2225 articles, and finally included a total of 1344 relevant articles for analysis. In order to ensure the consistency of the screening results, we trained 2 experts in the field of medical informatics (YH and J Liang) before the start of the study, and asked them to independently screen the same 20 randomly selected articles according to the inclusion and exclusion criteria. The results showed great consistency (kappa = 0.71), and the disagreement between the 2 experts was resolved by the third expert in the field of medical informatics (J Lei) through arbitration. Then, 2 experts (YH and J Liang) screened the remaining literature to ensure that the included literature was closely related to 5G medical applications. The detailed flow chart of literature screening is shown in Figure 1.

Inclusion criteria:

1. 5G technology is the main research subject or technology.
2. Application or analysis for specific medical scenarios.

Exclusion criteria:

1. Reviews, letters, errata, and comments.
2. Articles with incomplete information or full text cannot be obtained.
3. 5G technology is only used as a background introduction or future outlook, not the subject of research or the main technical solution.
4. There is no specific medical scenario, or the main application scenario of the technology is not in the medical field.

### 2.4. Data extraction and analysis

In terms of data extraction, we extracted the complete bibliographic information of the included literature, including title, author, author unit, publication time, keywords, abstract, publication name, and funding, and manually translated the bibliographic information of the Chinese literature into English. It is worth noting that we summarized Chinese literature and English literature with a correspondence address in China and conducted a preliminary comparative analysis with other English literature.

For data analysis, we used bibliometric methods to quantitatively analyze the characteristics of the included articles from the perspectives of publication trends, citations, core journals, core author groups, institutional cooperation, funding sources, etc,^[25,26]^ to understand the current research status of 5G technology applied in the medical field. Furthermore, this study used a 3-step pipeline framework to analyze hot topics. First, subject clustering is carried out on the high-frequency keywords included in the literature, and the main themes of relevant research and the relationship between the themes are analyzed through the keyword co-occurrence network. Second, the thematic map^[27]^ is used to analyze the relationship between the topics according to the density and centrality. Themes are clustered and mapped to explore current research hotspots. Finally, the evolution trend of topics related to 5G medical application research is analyzed from the time dimension.

For the selection of high-frequency keywords, we used Python software foundation (San Francisco) to preprocess all the keywords included in the literature (including converting Chinese to English, lowercasing English, removing stop words, and lemmatization). Then, according to the 80/20 rule,^[28]^ the threshold value of high-frequency keywords is calculated, and it is concluded that the word frequency is 5 times or more as high-frequency keywords.

Regarding the analysis tools used in the measurement, first, we used the bibliometrix package^[29,30]^ of R language (RStudio Inc., Boston) to analyze the literature. Then, we used the ggplot2 package^[31]^ to draw statistical graphs. Finally, we used VOSviewer (Centrum Voor Wetenschap en Technologische Studies, Leiden, The Netherlands)^[32]^ for network visualization. Furthermore, we performed data visualization in accordance with established guidelines.^[33,34]^

## 3. Results

### 3.1. Trend analysis of the number of articles

**Figure 2.**
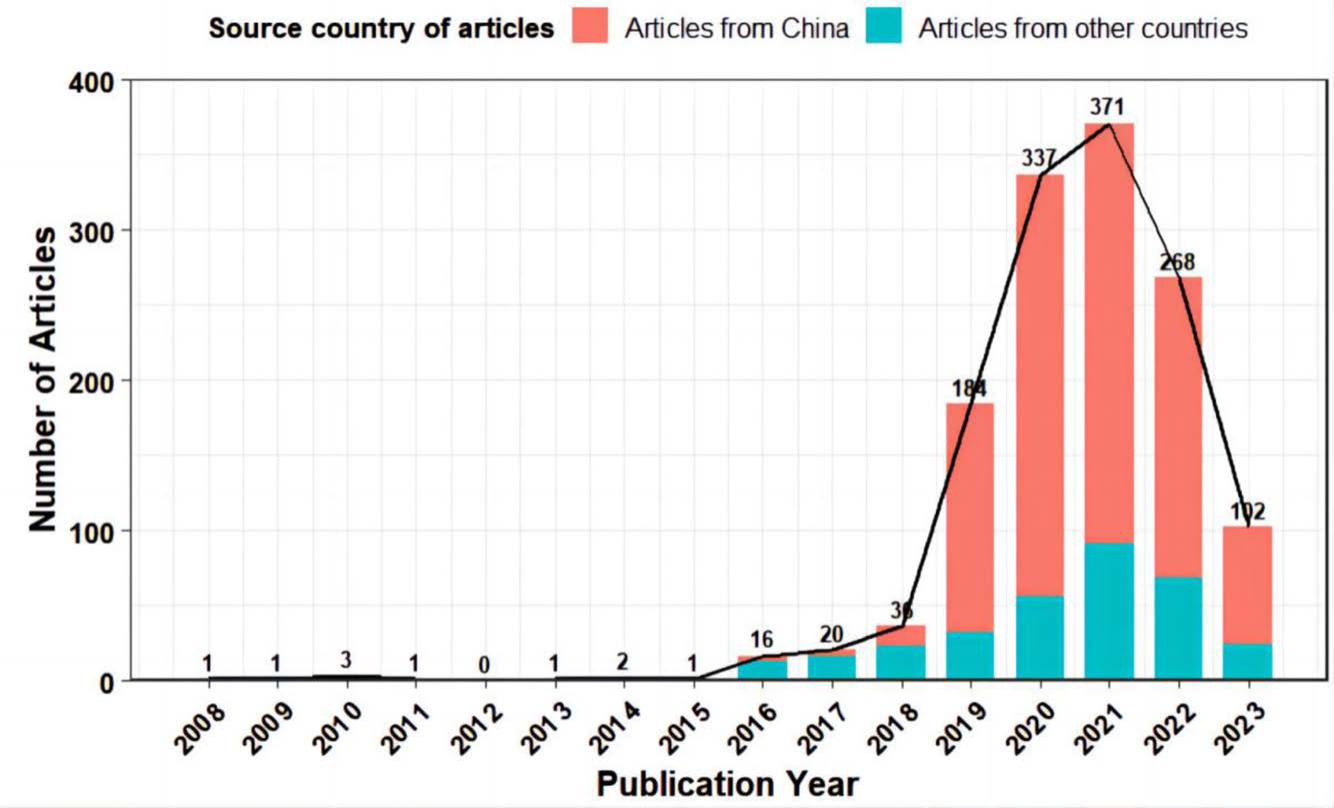
Number of articles changed over time.

Research on the application of 5G technology in the medical field has developed rapidly since 2016 and reached its peak in 2021. The number of relevant research in China (including Chinese literature and English literature with a correspondence address in China) far exceeds that of other countries. From 2008 to 2023, a total of 3354 authors published 1344 related articles in 706 different journals, of which 1014 were published by Chinese authors (75.45%) and 330 by authors from other countries (24.55%). According to the publication year of the literature, we drew a graph of the number of publications over time (Fig. 2) and found that the number of publications of relevant literature showed a trend of first increasing and then decreasing. Before 2016, the number of published articles in this field did not exceed 3 per year. After 2016, their number increased rapidly, reaching a peak (371 articles) in 2021, during which the average annual growth rate of articles was 352.56%. After 2021, the number of literature in this field began to decrease. Collectively, the publications received 5659 citations, yielding a mean citation rate of 4 citations per article.

### 3.2. Core journal analysis

The average quality of core journals in this field is low, and there is a lack of high-impact journals. Relevant research in China is mostly published in noncore journals, while most of the research in other countries is published in English journals included in the Science Citation Index, and the quality of the journals is higher than that in China. According to Bradford law in bibliometrics and related inferences,^[35]^ the number of core journals accounts for about 1/31 of the total number of journals. After calculation, the top 22 journals in this field by the number of published articles are core journals (Table A2, Supplemental Digital Content, https://links.lww.com/MD/P388), and their publications account for 23.51% of all articles. Among them, Chinese research is mostly published in 17 Chinese journals with a low degree of internationalization, while research from other countries is mostly published in 5 English journals included in Science Citation Index. The average impact factor of these 5 English journals in 2022 is 8, whose quality far exceeds that of Chinese journals. In addition, by drawing a comparative trend chart of the annual publications of the top 5 core journals (Figure A1, Supplemental Digital Content, https://links.lww.com/MD/P388), it was found that the number of publications in the 2 journals “*China Digital Medicine*” and “*China Hospital CEO*” fluctuated, while the number of publications in the other 3 core journals increased first and then decreased. The vast majority of Chinese journals started to pay attention to the application of 5G in the medical field in 2019, while the English journal “*IEEE Access*” has paid attention earlier and has published relevant literature since 2016. “*China Digital Medicine*” has the highest total number of publications and reached its peak in 2022 (n = 14). “*IEEE Access*” has been paying attention to 5G medical applications for the longest time and has published relevant literature for 6 years. However, except for “*China Digital Medicine*,” the remaining 4 core journals all reduced their focus on 5G medical applications after 2021.

### 3.3. Core authors analysis

Chinese authors have made far greater contributions to this field than other countries, but they published a small number of articles, their influence is relatively insufficient, and the cooperation among Chinese authors is poor. A total of 3354 authors participated in the relevant research, including 1344 first authors/corresponding authors. According to the Lotka law,^[36]^ there are 137 core authors in this field, and the articles published by these authors account for 36.61%. We judged the characteristics of core authors by analyzing the number of articles published by the top 10 authors and the dominance factor^[37]^ (the actual contribution of the author is measured by calculating the proportion of the first author/corresponding author in all the articles published by the author) (Figure A2, Supplemental Digital Content, https://links.lww.com/MD/P388). It was found that the top 10 core authors with the most published articles are all Chinese authors. Yuemei ranks highly in terms of published articles and dominance factor and has made the greatest contribution to the field of 5G medical applications. We also plotted the annual publication trends of the top 10 core authors based on the number of publications and the frequency of citations (Table A3, Supplemental Digital Content, https://links.lww.com/MD/P388). The size of the circle in the graph represents the annual publications, and the depth of the color represents the annual citations. The analysis found that 8 (80%) core authors started to publish relevant research in 2019. Yuemei, which contributed the most to this field, has a relatively short research year (2 years), and the number of publications (7 papers) and frequency of citations (0 times) are both small. Furthermore, no obvious collinear network was constructed among the core authors, suggesting poor collaboration among experts in the field.

### 3.4. Analysis of cooperation between countries and institutions

China is a country with the largest number of publications, and Chinese institutions such as China Mobile Communications Group Co., Ltd. and Zhejiang University are the main publishing institutions in this field. However, there is a lack of effective cooperation between countries and institutions. For the country of origin, 3354 authors come from 57 countries. We plotted the number of articles published by the top 10 most productive countries (Figure A3, Supplemental Digital Content, https://links.lww.com/MD/P388) and found that China has the largest number of relevant studies (n = 1014 [75.45%]), far exceeding India (n = 54 [4.02%]), the United States (n = 43 [3.20%]), and the United Kingdom (n = 27 [2.01%]). The cooperation between countries is poor, and the proportion of multiple-country publications in most countries is less than 15%. For publishing institutions, the top 5 institutions with the highest number of publishing articles are all Chinese institutions, namely China Mobile Communications Group Co., Ltd. (n = 48 [3.57%]), Zhejiang University (n = 40 [2.98%]), Chinese People’s Liberation Army General Hospital (n = 32 [2.38%]), China United Network Communications Group Co., Ltd. (n = 32 [2.38%]), and University of Electronic Science and Technology of China (n = 32 [2.38%]). We drew an institutional cooperation network diagram based on the author’s institutional data and found that no obvious clustering results could be constructed, indicating that cooperation between institutions in this field is relatively weak.

### 3.5. Funding analysis

China is the main funding country, with the Ministry of Science and Technology of China as the main funding agency. The number of funds in China far exceeds that of other countries, and the funding rate is relatively low. A total of 392 papers were funded by 705 funds, and the funding rate in this field was 29.17%, among which the funding rate of Chinese research was lower than that of other countries (25.94% vs 39.09%). Based on the literature fund, we drew a distribution map of the funding sources (Figure A4, Supplemental Digital Content, https://links.lww.com/MD/P388), and the results show that a total of 33 countries/regions have provided funding for 5G medical application research. China (531 [75.32%]), the European Union (36 [5.11%]), and the United States (23 [3.26%]) are the main funding countries/regions, and the number of Chinese funds far exceeds that of other countries. In addition, a total of 243 institutions provide funding support, and 7 institutions have funded no less than 10 projects (Table A4, Supplemental Digital Content, https://links.lww.com/MD/P388). Among them, the Ministry of Science and Technology of China (69 [9.79%]) provided the most funding support, followed by the National Natural Science Foundation of China (66 [9.36%]) and the European Commission (36 [5.11%]). It is worth noting that among the funded projects from Chinese institutions, there are 46 (8.66%) national key R&D projects, covering multiple fields such as medical care, communications, and military industry, which shows that China attaches great importance to research on 5G applications.

### 3.6. Hot topic analysis

#### 3.6.1. High-frequency keywords cluster analysis

**Figure 3.**
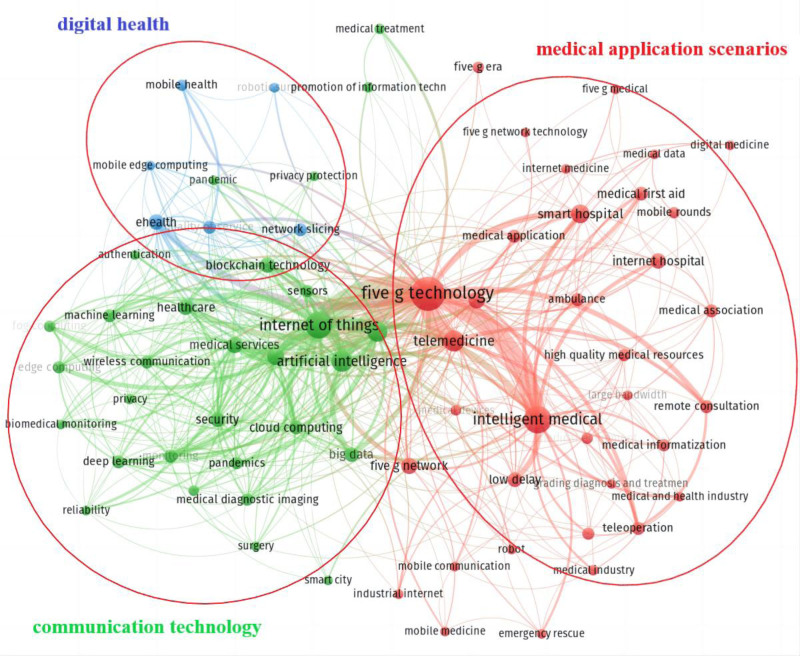
High-frequency keywords co-occurrence network diagram.

According to the high-frequency keyword co-occurrence network diagram (Fig. 3), research topics in this field are clustered into 3 categories: medical application scenarios, communication technology, and digital health. Among them, the red category represents specific medical scenarios for 5G applications (with 36 keywords), such as telemedicine, internet hospital, ambulance, and smart hospital. Among them, Chinese research focuses on the construction of internet hospitals, while other countries focus more on telemedicine. The green category (30 keywords) represents communication technology and artificial intelligence technology, including Internet of Medical Things (IoMT), cloud computing, wearable devices, and machine learning. The blue category (6 keywords) represents digital health, such as mobile health and electronic health. For the concept of connection between categories, the concept between medical application scenarios and communication technology is mainly in medical specialties, such as surgery and medical imaging. Between communication technology and digital health is digital technologies such as blockchain and identity verification. The concept between medical application scenarios and digital health is mainly the digitization of medical scenarios, including privacy protection, information protection, etc.

The high-frequency keywords in 330 studies from other countries are not enough to construct an effective co-occurrence network, indicating that the current research from other countries lacks concentration.

#### 3.6.2. Thematic map analysis

**Figure 4.**
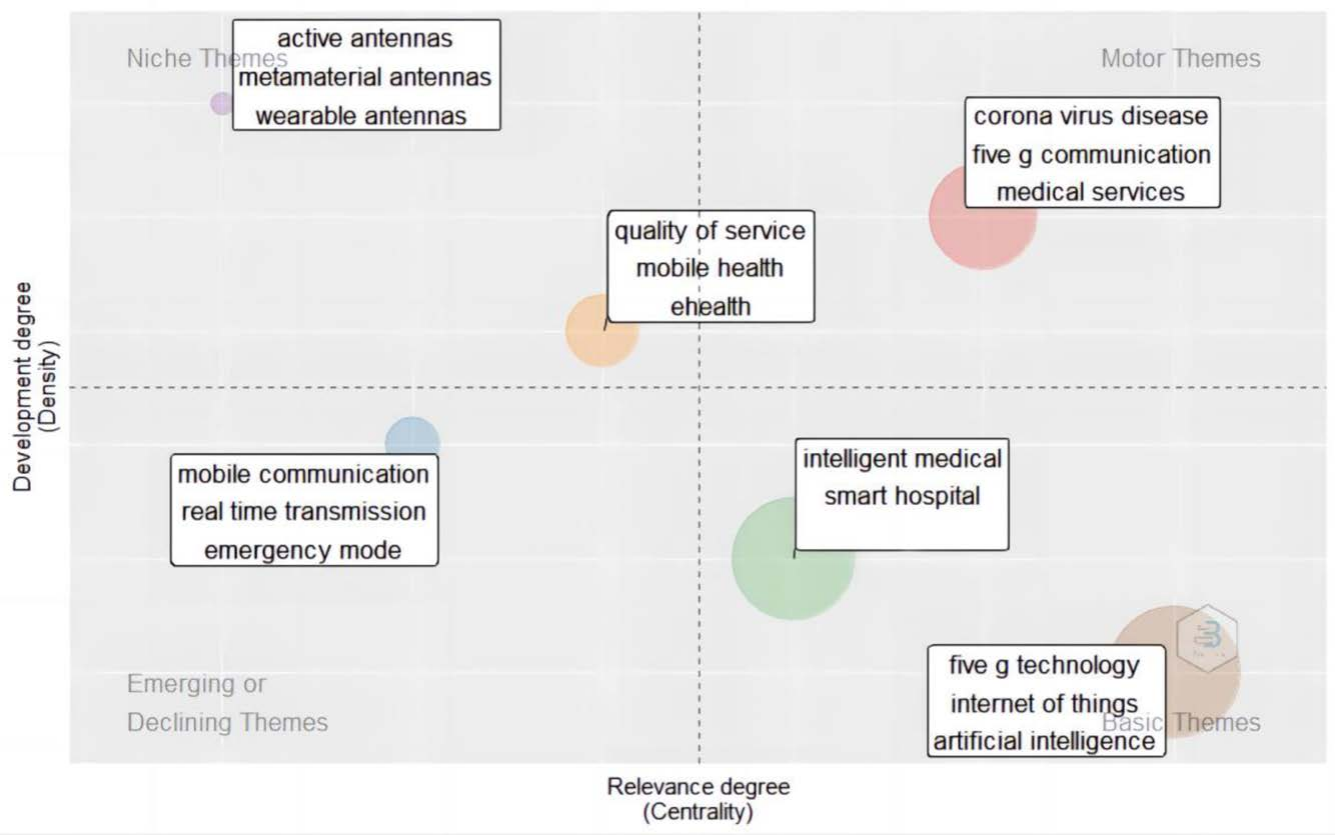
Domain theme map.

We calculated the density and centrality of the high-frequency keyword co-occurrence matrix, visualized 2-dimensionally the main categories, and drew a theme map that reflects the research’s popularity and importance (Fig. 4). It can be seen from the figure that the first quadrant includes the COVID-19, 5G communication and medical services, indicating that the 5G smart medical service for the COVID-19 is a hot topic in this field recently and is of high importance. The second quadrant mainly includes mobile health, wearable devices, indicating that although the research on 5G transmission support technology is very mature, it is less important because it is at the bottom of data analysis. The third quadrant includes mobile communication and real-time transmission, indicating that 5G real-time communication in medical scenarios is an edge topic. The fourth quadrant includes not only new technologies, such as 5G technology, the IoMT, and artificial intelligence, but also medical application scenarios, such as smart medical care and smart hospitals, indicating that the construction of smart hospitals based on new technologies such as the IoMT may be a potential hot spot in the future.

#### 3.6.3. Topic evolution trend analysis

**Figure 5.**
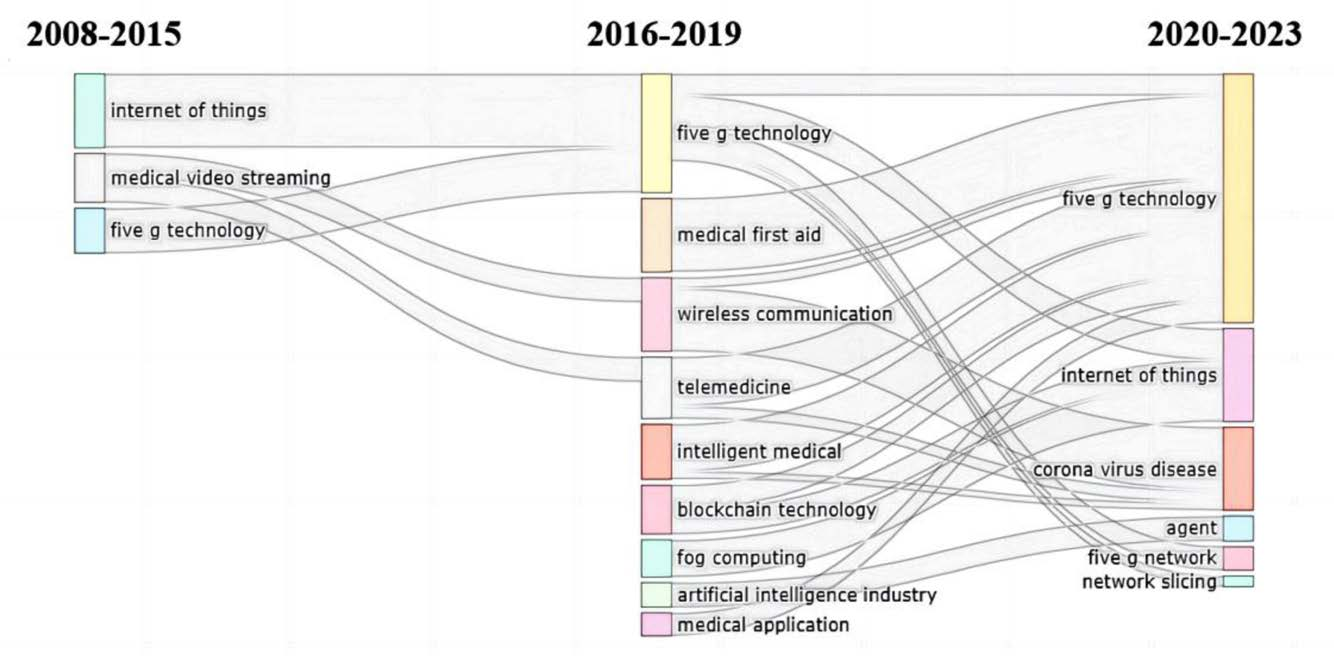
2008 to 2023 Sankey diagram of research topic evolution trends.

By exploring the evolution trend of the topic through the Sankey diagram, we found that the development of research topics in this field conforms to the entire process of data analysis, from the data acquisition layer such as wearable devices to the data transmission layer such as blockchain to the data application layer such as internet hospitals. We incorporated the dimension of time into the high-frequency keyword co-occurrence matrix and topic clustering to analyze the topic evolution from 2008 to 2023 (Fig. 5). It was found that 5G technology runs through and is the technical basis of all research. From 2008 to 2015, the number of relevant studies was very small. At this time, medical institutions initially realized informatization. Researchers mainly focused on the data acquisition layer, using wearable devices to obtain medical data and applying 5G technology to quickly analyze the obtained medical data. From 2016 to 2019, the number of related research increased year by year. Researchers mainly focused on the data transmission layer, applying new communication technologies (such as blockchain, cloud computing, and IoMT) to different medical scenarios (such as medical emergency, telemedicine, and Internet hospital). From 2020 to 2023, related research was saturated gradually and began to decrease. Researchers mainly focus on the data application layer. Due to the impact of COVID-19, researchers have focused on the application of 5G networks in internet hospitals during this period. In addition, it was found that combining new technologies such as 5G technology, the IoMT, and artificial intelligence to build a smart hospital system, optimize the process of smart medical services, and carry out smart patient services is a potential research trend in the future. In the transition phase from Internet hospitals to smart hospitals, most of the research has gradually shifted from the construction of theoretical frameworks to the research and development of physical smart medical systems, which have not only realized online medical services but also further developed intelligent medical services.

## 4. Discussion

Through a systematic literature search and screening, this study included a total of 1344 Chinese and English literature on the application of 5G in the medical field as of August 4, 2023. We used bibliometrics to quantitatively analyze the publication characteristics of the included literature and found that for the number of publications, the research on the application of 5G technology in the medical field has developed rapidly since 2016 and reached its peak in 2021. For research quality, this research area shows below-average citation frequency per article. Meanwhile, based on the journal impact factors and the zoning of the journal where the article is located, it is found that the impact factors of core journals in this field are relatively low, and the average research quality is poor. The influence of core journals and research quality in China need to be further improved. For the core authors, Chinese authors are the main contributors in this field, but there is a lack of leaders and effective cooperation among authors. For publishing/funding countries and institutions, China is the country with the largest number of articles and funds. China Mobile Communications Group Co., Ltd. is the main publishing agency, while the Ministry of Science and Technology of China is the main funding agency. For hot topics, the development of research topics in this field follows the trend of “data acquisition layer”-“data transmission layer”-“data application layer,” and people are paying more and more attention to the application of new communication technologies such as blockchain, cloud computing, and IoMT in digital medical scenarios, such as medical emergency, remote applications, medical care, and Internet hospitals.

### 4.1. China-related research far exceeds that of other countries, but its quality is low

The number of studies in China (including Chinese and English literature with a mailing address in China) far exceeds that of other countries, but the quality of its articles is lower, which is reflected in the lack of highly influential articles and core journals. Research on the application of 5G in the medical field was first published in 2008, increased rapidly after 2016, and reached its peak in 2021 (n = 371), but there has been a downward trend since then, which may be related to the widespread application of 5G in hospitals during the COVID-19. Telemedicine and 5G developed rapidly during the COVID-19, but their attention continued to decline after the COVID-19. The number of publications by Chinese authors (n = 1014 [75.45%]) far exceeds that of other countries (n = 330 [24.55%]). This may be related to the Chinese government’s policy for 5G+ medical and health applications and the expansion of the Internet medical and health market. For government policy support, in 2016, the General Office of the State Council of China issued the “Guiding Opinions on Promoting and Standardizing the Development of Big Data Applications in Health and Medical Care”^[38]^ to comprehensively establish a telemedicine application system. In 2018, the “Opinions on Promoting the Development of Internet + Medical and Health”^[39]^ began to promote the development of Internet + medical and health services. In 2020, the National Health Commission issued the “Opinions on Strengthening the Construction of a National Health Information Standardization System”^[40]^ to further strengthen the application of 5G technology in the medical field. In 2021, the “5G Application Sail Action Plan (2021–2023)”^[41]^ released by the Ministry of Industry and Information Technology detailed the medical scenarios of 5G technology applications, such as 5G medical robots, 5G ambulances, and a remote diagnosis and treatment platform. For market size, in 2023, the size of China’s Internet medical market is expected to exceed 49.9 billion USD, the number of digital medical platform users may increase to 882 million,^[42]^ the number of Internet diagnosis and treatment in hospitals may increase 17 times year-on-year, and the number of prescriptions may increase by nearly 10 times.^[43]^ The policy support of the Chinese government and the increasing demand in the internet medical market have promoted the rapid growth of 5G medical application research in China. Furthermore, the COVID-19 outbreak has accelerated this growth. However, based on the impact factors and partitions of the journal in which the article is located, as well as the number of citations, the quality of the relevant research is poor, mainly reflected in the low impact factors of the journal, and the article is cited less. About 94.12% of the published journals are not highly internationalized, and most of the articles have been cited less than once. It is worth noting that the influence of Chinese-related journals is lower, and articles are cited less frequently. For example, most of China’s research has been published in 17 Chinese journals with lower levels of internationalization, and most articles have not even been cited once. It is recommended that Chinese scholars expand their international perspective, combine field needs and cutting-edge hot topics, and publish articles with theoretical and practical value in influential international journals to gain more citations and recognition from other scholars. In addition, Chinese research has the largest amount of funding (75.32%), which may be related to China’s unique medical environment and system. There is a large rural population among Chinese residents, and the basic medical facilities are not complete, which prompts regional funds to tend to support the construction of basic medical institutions. At the same time, China is implementing the medical system of medical consortia and medical communities, which means that well-developed large hospitals collaborate to manage multiple grassroots medical institutions through communication technologies such as 5G and improve their medical service capabilities.

### 4.2. Lack of effective collaboration between authors, countries, and institutions

China is the main funding country, and Chinese authors are the main contributor group, but international cooperation is poor, and there is a lack of effective cooperation between authors and institutions. China has provided a total of 531 (75.32%) funds to 263 (25.94%) research and 85.71% of the funding institutions are from China. Chinese authors published the most articles, and the top 10 core authors with the most published articles are all from China. However, the international cooperation is poor, and the proportion of multiple-country publications in most countries is less than 15% and no obvious cooperation network has been established between the core authors and institutions, which shows that experts and institutions in this field focus on independent research and lack cooperation and communication. This may be related to the differences in countries’ attention to 5G technology and the confidentiality of medical data. For policy support, China has the strongest support for 5G technology and has rapidly promoted the construction of 5G network infrastructure.^[44]^ However, other countries have not paid much attention to 5G technology,^[45]^ making few authors choose to cooperate cross-borderly to explore 5G technology. For data confidentiality, medical and health data, especially hospital patient data, have strong privacy and are rarely interconnected in different hospitals.^[46]^ Therefore, in order to ensure the security of medical data, hospitals seldom cooperate with other institutions. In addition, there is a lack of clear clusters for cooperation between countries and institutions, which can also be explained as the fair distribution of support opportunities in this field, rather than the concentration and polarization of funds.

### 4.3. Deep integration of 5G with wearable devices and artificial intelligence technology, but attention should be paid to issues such as data privacy

5G is developing rapidly in the field of wireless healthcare, with its core technology being 5G communication networks, which have been deeply integrated with wearable devices and artificial intelligence technology, and is mostly used in scenarios such as telemedicine, mobile health, and smart hospital. However, further attention is needed to address issues such as data privacy breaches, limited data monitoring coverage, low reliability of 5G communication networks, poor data quality, and inability to transmit data in real-time. For the current development status of 5G technology, most scholars focus on the construction of 5G communication networks and apply them in the medical field. For example, Guo and Li^[16]^ systematically analyzed the current status of 5G communication network construction in the field of personalized nursing in China and summarized its application prospects. Sensors and wearable devices are important tools to help obtain health data. They obtain patient physiological signal data through invasive or noninvasive means and achieve rapid processing and dissemination of health data through 5G communication networks. In addition, with the development of information technology, intelligent decision support has become a hot topic in the medical field, and 5G technology is gradually deeply integrated with various artificial intelligence technologies, such as machine learning and deep learning. These artificial intelligence technologies help patients and doctors make preliminary medical judgments through data preprocessing and algorithm construction. For the application scenarios of 5G technology, most scholars combine technologies such as the IoMT and blockchain to apply 5G to the construction of telemedicine, mobile health, and smart hospitals. For example, Liang et al^[47]^ designed a remote medical robot for abdominal ultrasound examination by combining 5G technology and robotics technology. Tang et al^[48]^ designed a smart hospital system based on 5G technology and conducted on-site experiments in physical hospitals. For the existing problems and improvement suggestions of 5G technology, there are still issues such as data privacy leakage, low reliability of 5G communication networks, and inability to transmit data in real time. This article suggests strengthening the desensitization of medical data, promoting the application of blockchain technology, transfer learning, and federated learning in the medical field, and improving the security and privacy protection of medical data. Meanwhile, it is recommended that hospitals build 5G communication networks based on actual medical scenarios and enhance the communication efficiency of medical data by combining technologies such as the IoMT.

### 4.4. The construction of smart hospitals based on the IoMT may become a new research hotspot

The development of research topics follows the trend of “data acquisition layer”-“data transmission layer”-“data application layer.” The construction of 5G internet hospitals is a research hotspot during COVID-19, and the construction of smart hospitals based on new technologies such as the IoMT may be a potential hotspot in the future. At present, the topics in this field are gathered into 3 categories: medical application scenarios, communication technology, and digital health. Among them, smart hospitals, telemedicine, and Internet hospitals are the main application scenarios; the IoMT, wearable devices, blockchain, and cloud computing are the main communication technologies; and electronic health and mobile health are the main digital medical scenarios. Research in this field runs through the entire path of data analysis, obtaining health data from wearable devices, using 5G technology to realize real-time transmission of data, and then realizing the deployment and application of data throughout the hospital through technologies such as the IoMT. For hot topics, 5G smart medical services in Internet hospitals are a hot research topic during the COVID-19 and have a good research foundation. For example, Dacan et al^[49]^ used case analysis to explore the key elements of the 5G smart medical service system in the prevention and control of COVID-19. The full life cycle management of health data based on wearable devices is a current research hotspot. For example, Miao et al^[50]^ systematically summarized the latest developments in wearable technologies related to cardiovascular care, outlined the challenges encountered, and proposed solutions. For the diseases involved in the study, 5G technology not only accelerated the process and efficiency of COVID-19 management in the hospital but also promoted the management of chronic diseases, such as independent health management of hypertension and diabetes. Users can monitor chronic disease indicators and intervene in behavior through wearable devices and mobile health apps. In addition, 5G technology has further promoted the health management of obesity, sleep disorders, and mental illnesses, achieving the interconnection of personal health data through technologies such as the IoMT, and combining artificial intelligence algorithms to achieve early warning of sub health status. In addition, Chinese studies focus on the construction of internet hospitals,^[51,52]^ while other countries focus more on telemedicine.^[53,54]^ For future research trends, the construction of smart hospitals based on the IoMT, blockchain, and cloud computing may be a potential hot topic in the future. The smart hospital system includes 3 major areas: electronic medical records for medical staff, smart services for patients, and smart management for hospitals. Researchers can use IoMT to realize data exchange and behavioral interaction among medical staff, patients, medical institutions, and equipment, thereby improving medical efficiency, reducing medical errors, and saving medical costs. For research topics, it is recommended that researchers conduct relevant research on electronic medical records, patient intelligent services, and hospital intelligent management under the application of the IoMT and 5G technology.^[55–59]^ For practical suggestions, it is recommended that management personnel focus on the construction of intelligent hospitals based on IoMT, including smart hospitals, telemedicine, and Internet hospitals.^[60,61]^ For data analysis, it is recommended that researchers focus on the entire process of 5G medical data analysis, including the acquisition, real-time transmission, processing, and application of medical data.^[62]^

Beyond academic contributions, this study offers practical guidance for stakeholders. For researchers, it is recommended that they prioritize interdisciplinary collaborations to address technical gaps (e.g., data privacy and AI integration). For policymakers, we recommend that they standardize medical data privacy rules to protect patient information, regulate 5G use in hospitals, and create incentives for 5G adoption in smart hospitals. For industry professionals, they can leverage our trend analysis to develop IoMT-based smart hospital solutions. These coordinated efforts will accelerate the translation of 5G technologies into equitable, efficient healthcare systems.

### 4.5. Limitations

This study has some limitations. For scientific databases, as a bibliometric analysis, we focused solely on journal articles and conference proceedings to ensure research quality. However, future studies can incorporate additional sources such as monographs, patents, and policy documents to enhance the comprehensiveness and generalizability of findings.^[63]^ For search terms, we systematically identified relevant keywords based on previously published literature and employed fuzzy search techniques to improve recall. However, a small subset of articles may have used unconventional terminology. We addressed this through cross-validation methods. For analytical metrics, future research can incorporate additional metrics such as co-citation analysis or burst terms, supplemented by more diverse visualizations (e.g., technology evolution trend graphs) for richer insights. For analysis content, as a bibliometric study, it can explore the overall research progress in the field of 5G medical applications through quantitative analysis, but it cannot conduct more in-depth content mining and lacks detailed content analysis of hot topics in the field. In addition, considering that the main group of publishers in this field is Chinese authors, high-frequency keywords in other countries’ literature cannot be effectively clustered. Therefore, we are unable to make a detailed comparison of the differences between research topics in China and other countries, and future research can further explore this from the perspective of content analysis.

## 5. Conclusions

This study uses bibliometrics to quantitatively analyze 1344 articles on the application of 5G technology in the medical field. It was found that research in this field has developed rapidly since 2016, but there has been a downward trend in the past 2 years. China provides the most funding and publishes far more relevant research than other countries, but the quality of the articles is low and most of them are published in journals with low impact. Chinese authors are the main contributor group in this field, but their influence is low and international cooperation is insufficient. Most studies have reported that 5G technology can alleviate problems such as insufficient medical resources, uneven distribution of medical resources, and difficulties in remote medical treatment. It promotes rational distribution of high-quality medical resources to primary health terminals and improves regional medical level. Researchers should combine the development characteristics of communication technologies in different countries, actively utilize public medical datasets while ensuring data privacy and security measures, and explore the application of 5G technology in medical scenarios in various countries. In addition, the research topics have also undergone major changes. Research hotspots have gradually shifted from the construction of 5G internet hospitals during the COVID-19 to the construction of smart hospitals based on the IoMT. The research focus has shifted from the data transmission layer, such as wearable devices, to the data application layer, such as internet hospitals. Although different countries used 5G technology to improve medical services, more comprehensive content analysis is needed to explore the differences in the development status and application scenarios of 5G medical technology in various countries.

## Author contributions

**Data curation:** Yunfan He, Jun Xie, Zheyang Weng, Jun Liang.

**Formal analysis:** Yunfan He.

**Investigation:** Yunfan He.

**Methodology:** Yunfan He.

**Software:** Yunfan He.

**Supervision:** Yunfan He, Jun Liang, Jianbo Lei.

**Visualization:** Yunfan He.

**Writing – original draft:** Yunfan He.

**Writing – review & editing:** Yunfan He, Jun Xie, Zheyang Weng, Fangyu Yang, Yutao Wei, Jun Liang, Jianbo Lei.

**Funding acquisition:** Jun Liang, Jianbo Lei.

**Validation:** Jun Liang.

**Conceptualization:** Jianbo Lei.

## Supplementary Material


