# Supplementary material for: Exploring the emerging trends and hot topics of 5G technology application in wireless medicine: A bibliometric and visualization analysis
Source: Medicine (Baltimore). 2025 Jul 18;104(29):e43310. doi: 10.1097/MD.0000000000043310 (PMC12282767; doi:10.1097/MD.0000000000043310)
Supplement: Supplementary file 1 [file medi-104-e43310-s001.docx]

# Appendix A

**For Manuscript:**

**“Exploring the emerging trends and hot topics of 5G technology application in medicine: a bibliometric and visualization analysis”**

Contents

[Appendix A 1](#_Toc10113)

[1 Summary of Tables 1](#_Toc24958)

[2 Summary of Figures 5](#_Toc27898)

# 1 Summary of Tables

**Table A1.** Specific search term and search result of different literature databases.

| **Search database** | **Search formula** | **Search result** |
| --- | --- | --- |
| Web of Science Core Collection | TS=(5G OR "5th generation mobile communication technology" OR "5th generation mobile network" OR "5th generation wireless system" OR "5th-generation") AND TS=(medicine OR medical OR witmed OR "wise information technology of med" OR "smart hospital" OR "internet hospital" OR "hospital informatization" OR "hospital management" OR "mobile health" OR "digital health" OR "wearable device") | 947 |
| PubMed | (5G[Title/Abstract]) OR "5th generation mobile communication technology"[Title/Abstract] OR "5th generation mobile network"[Title/Abstract] OR "5th generation wireless system"[Title/Abstract] OR "5th-generation"[Title/Abstract]) AND (medicine[MeSH Terms] OR medical[Title/Abstract] OR witmed[Title/Abstract] OR "wise information technology of med"[Title/Abstract] OR "smart hospital"[Title/Abstract] OR "internet hospital"[Title/Abstract] OR "hospital informatization"[Title/Abstract] OR "hospital management"[Title/Abstract] OR "mobile health"[Title/Abstract] OR "digital health"[Title/Abstract] OR "wearable device"[Title/Abstract]) | 400 |
| Embase | (5g:ab,ti OR '5th generation mobile communication technology':ab,ti OR '5th generation mobile network':ab,ti OR '5th generation wireless system':ab,ti OR '5th-generation':ab,ti) AND (medicine:ab,ti OR medical:ab,ti OR witmed:ab,ti OR 'wise information technology of med':ab,ti OR 'smart hospital':ab,ti OR 'internet hospital':ab,ti OR 'hospital informatization':ab,ti OR 'hospital management':ab,ti OR 'mobile health':ab,ti OR 'digital health':ab,ti OR 'wearable device':ab,ti) | 395 |
| IEEE | (5G OR "5th generation mobile communication technology" OR "5th generation mobile network" OR "5th generation wireless system" OR "5th-generation") AND (medicine OR medical OR witmed OR "wise information technology of med" OR "smart hospital" OR "internet hospital" OR "hospital informatization" OR "hospital management" OR "mobile health" OR "digital health" OR "wearable device") | 829 |
| CNKI | (SU='5G' OR SU='第五代移动通信技术' OR SU='第五代移动通信网络') AND (SU='医学' OR SU='医疗' OR SU='智慧医院' OR SU='互联网医院' OR SU='医院信息化' OR SU='医院管理' OR SU='急诊急救' OR SU='辅助诊疗' OR SU='移动健康' OR SU='数字健康' OR SU='可穿戴设备') | 1,174 |
| Wanfang | 主题:("5G" or "第五代移动通信技术" or "第五代移动通信网络") and 主题:("医学" or "医疗" or "智慧医院" or "互联网医院" or "医院信息化" or "医院管理" or "急诊急救" or "辅助诊疗" or "移动健康" or "数字健康" or "可穿戴设备") | 1,647 |
| VIP | M=("5G" OR "第五代移动通信技术" OR "第五代移动通信网络") AND M=("医学" OR "医疗" OR "智慧医院" OR "互联网医院" OR "医院信息化" OR "医院管理" OR "急诊急救" OR "辅助诊疗" OR "移动健康" OR "数字健康" OR "可穿戴设备") | 620 |
| Summary |  | 6,012 |

**Table A2.** Core journal articles, quality, JCR category and impact factor.

| **Rank** | **Source** | **Articles** | **Journal quality** | **JCR category** | **JCR partition** | **2022 JIF** |
| --- | --- | --- | --- | --- | --- | --- |
| 1 | China digital medicine | 32 | Non-core | NULL | NULL | NULL |
| 2 | IEEE access | 31 | SCI | COMPUTER SCIENCE, INFORMATION SYSTEMS | Q2 | 3.9 |
| 3 | Chinese hospital director | 31 | Non-core | NULL | NULL | NULL |
| 4 | Communication world | 21 | Non-core | NULL | NULL | NULL |
| 5 | Journal of medical informatics | 21 | Non-core | NULL | NULL | NULL |
| 6 | China new news | 19 | Non-core | NULL | NULL | NULL |
| 7 | Shanghai informatization | 13 | Non-core | NULL | NULL | NULL |
| 8 | Communications and information technology | 12 | Non-core | NULL | NULL | NULL |
| 9 | Chinese journal of emergency medicine | 12 | Core chinese | NULL | NULL | NULL |
| 10 | IEEE transactions on industrial informatics | 11 | SCI | AUTOMATION & CONTROL SYSTEMS | Q1 | 12.3 |
| 11 | Digital communication world | 11 | Non-core | NULL | NULL | NULL |
| 12 | Modern health care | 11 | Non-core | NULL | NULL | NULL |
| 13 | IEEE network | 10 | SCI | COMPUTER SCIENCE, HARDWARE & ARCHITECTURE | Q1 | 9.3 |
| 14 | Sensors | 10 | SCI | CHEMISTRY, ANALYTICAL | Q2 | 3.9 |
| 15 | Communication world | 10 | Non-core | NULL | NULL | NULL |
| 16 | Chinese journal of health information management | 10 | Non-core | NULL | NULL | NULL |
| 17 | Communications management and technology | 9 | Non-core | NULL | NULL | NULL |
| 18 | Continuing medical education in China | 9 | Non-core | NULL | NULL | NULL |
| 19 | Chinese medical equipment | 9 | Non-core | NULL | NULL | NULL |
| 20 | IEEE internet of things journal | 8 | SCI | COMPUTER SCIENCE, INFORMATION SYSTEMS | Q1 | 10.6 |
| 21 | Economy | 8 | Non-core | NULL | NULL | NULL |
| 22 | Chinese medical equipment | 8 | Non-core | NULL | NULL | NULL |

**Table A3.** Number of publications and Dominance Factor ranking of the top 10 authors.

| **Author** | **Articles number** | **Dominance factor** | **Ranking of published articles** | **Ranking of dominance factor** |
| --- | --- | --- | --- | --- |
| LIU YANG | 8 | 0.75 | 1 | 5 |
| YUEMEI | 7 | 1 | 2 | 1 |
| ZHANG QI | 7 | 0.14 | 2 | 8 |
| ZHANG YEJIANG | 7 | 0 | 2 | 9 |
| LIU LI | 6 | 0.83 | 5 | 3 |
| LV FAQIN | 6 | 0.33 | 5 | 7 |
| WANG C | 6 | 0 | 5 | 9 |
| FENG YI | 5 | 0.40 | 8 | 6 |
| GUO XIAOYA | 5 | 1 | 8 | 1 |
| HE PING | 5 | 0.80 | 8 | 4 |

**Table A4.** Institutions with no less than 10 funds.

| **Institution** | **Number of funding** | **Proportion** |
| --- | --- | --- |
| Ministry of Science and Technology of China | 69 | 9.79% |
| National Natural Science Foundation of China | 66 | 9.36% |
| European Commission | 36 | 5.11% |
| Ministry of Education of China | 57 | 8.09% |
| Science and Technology Agency in Sichuan | 20 | 2.84% |
| Science and Technology Commission of Shanghai | 14 | 1.99% |
| Science and Technology Commission of Beijing | 10 | 1.42% |

# 2 Summary of Figures


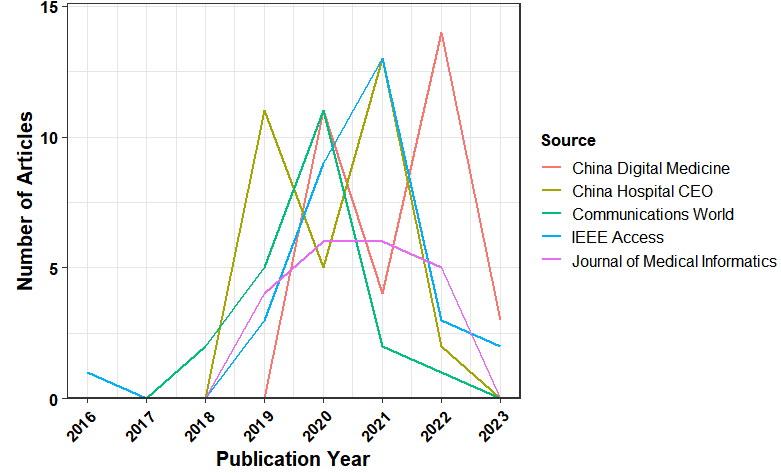


**Figure A1.** Comparison trend chart of the annual publications of the top 5 core journals.


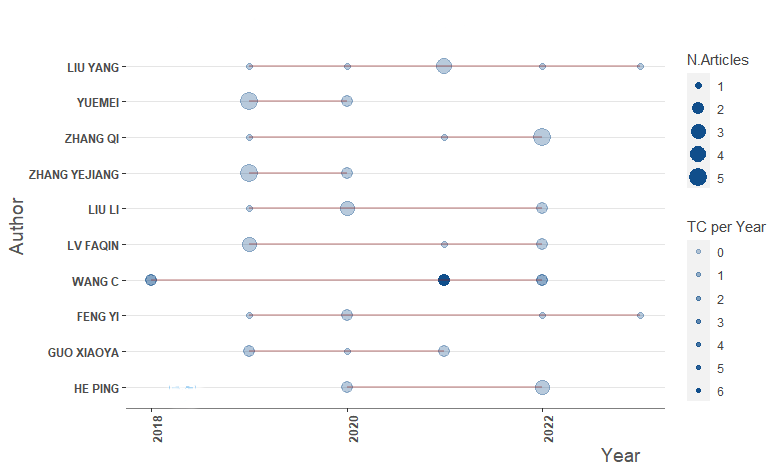


**Figure A2.** Publishing trends of the top 10 authors.


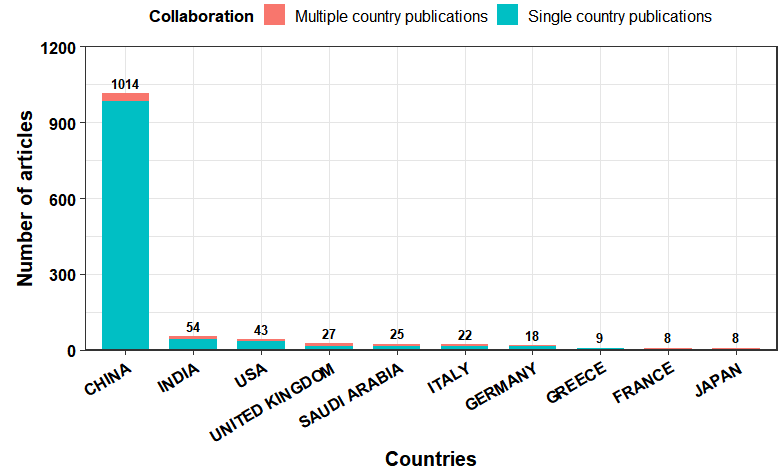


**Figure A3.** Distribution of the number of individual and cooperative publications of the top 10 high-yield countries.

Note: We define the country where the communication unit of an article is located as the publishing country of the article.


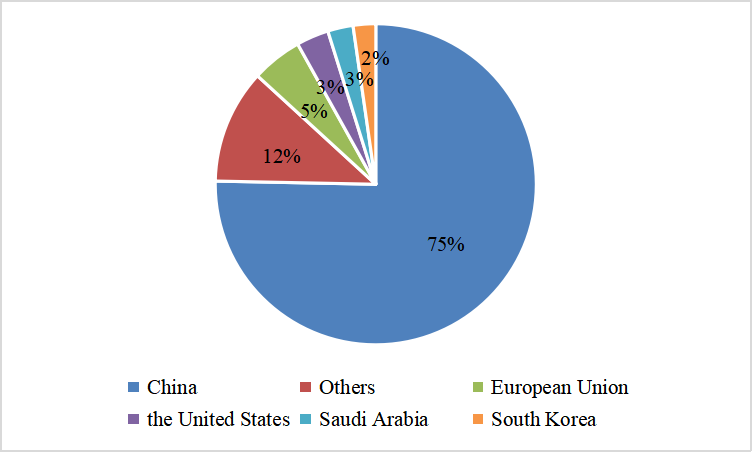


**Figure A4.** Funds source country distribution map.
